# Retrospective review of the prevalence and risk factors of anaemia among antenatal mothers attending health clinics in Alor Gajah, Melaka

**DOI:** 10.51866/oa.135

**Published:** 2022-11-25

**Authors:** Norsiah Ali, Zahratul Nur Kalmi, Nadya Sufia Sanusi, Azaria Ahad, Noor Asyiela Mohd Khairuddin, Sakinah Raain Rosman, Fazlina Rosli, Salbiah Mohd, Hannan Ismail, Norazimah Zainal, Mariany Ali, Kamsiah Salleh, Zaharah Razali, Haniah Abu Bakar, Azlina Jahaya, Noorhafizan Johar, Norhasiah Mamat, Siti Suhaila Ab Hamid, Nadia Bari, Noraziah Abd Rahman, Ezra Mohammad

**Affiliations:** 1 MD (USM), Masters in Family Medicine (UM), Fellowship in Addiction Medicine (Manash) Family Medicine Consultant, Klinik Kesihatan Masjid Tanah, Alor Gajah, Melaka, Malaysia. Email: norsiahrahim@yahoo.com.my; 2 B. Sc (Nutrition), M. Sc. (Community Nutrition), Nutritionist Pejabat Kesihatan Daerah Alor Gajah, Alor Gajah, Melaka, Malaysia.; 3 Medical Officer, Pejabat Kesihatan Daerah Alor Gajah, Alor Gajah, Melaka, Malaysia.; 4 Medical Officer, Pejabat Kesihatan Daerah Alor Gajah, Alor Gajah, Melaka, Malaysia.; 5 Medical Officer, Pejabat Kesihatan Daerah Alor Gajah, Alor Gajah, Melaka, Malaysia.; 6 Medical Officer, Pejabat Kesihatan Daerah Alor Gajah, Alor Gajah, Melaka, Malaysia.; 7 Medical Officer, Pejabat Kesihatan Daerah Alor Gajah, Alor Gajah, Melaka, Malaysia.; 8 B. Sc (Nutrition), Nutritionist, Pejabat Kesihatan Daerah Alor Gajah, Alor Gajah, Melaka, Malaysia.; 9 B. Sc (Nutrition), Nutritionist, Pejabat Kesihatan Daerah Alor Gajah, Alor Gajah, Melaka, Malaysia.; 10 Nurse, Pejabat Kesihatan Daerah Alor Gajah, Alor Gajah, Melaka, Malaysia.; 11 Nurse, Pejabat Kesihatan Daerah Alor Gajah, Alor Gajah, Melaka, Malaysia.; 12 Nurse, Pejabat Kesihatan Daerah Alor Gajah, Alor Gajah, Melaka, Malaysia.; 13 Nurse, Pejabat Kesihatan Daerah Alor Gajah, Alor Gajah, Melaka, Malaysia.; 14 Nurse, Pejabat Kesihatan Daerah Alor Gajah, Alor Gajah, Melaka, Malaysia.; 15 Nurse, Pejabat Kesihatan Daerah Alor Gajah, Alor Gajah, Melaka, Malaysia.; 16 Nurse, Pejabat Kesihatan Daerah Alor Gajah, Alor Gajah, Melaka, Malaysia.; 17 Nurse, Pejabat Kesihatan Daerah Alor Gajah, Alor Gajah, Melaka, Malaysia.; 18 Nurse, Pejabat Kesihatan Daerah Alor Gajah, Alor Gajah, Melaka, Malaysia.; 19 Family Medicine Specialist, Pejabat Kesihatan Daerah Alor Gajah, Alor Gajah, Melaka, Malaysia.; 20 Family Medicine Specialist, Pejabat Kesihatan Daerah Alor Gajah, Alor Gajah, Melaka, Malaysia.; 21 Family Medicine Specialist, Pejabat Kesihatan Daerah Alor Gajah, Alor Gajah, Melaka, Malaysia.

**Keywords:** Anaemia in pregnancy, Risk factors of anaemia, Prevalence of anaemia

## Abstract

**Introduction:**

Anaemia is common during pregnancy and can lead to miscarriage, intrauterine growth retardation, premature labour and antepartum haemorrhage. Anaemia in pregnancy is defined as a haemoglobin (Hb) level of <11 g/dL.

**Method:**

This retrospective review included 407 antenatal mothers diagnosed with anaemia at 36±1 weeks of gestation at all 10 health clinics in Alor Gajah between January and December 2018.

**Results:**

According to the district annual returns, 2,407 antenatal mothers (36 weeks of gestation) were registered in the health clinics in Alor Gajah in 2018. Among them, the prevalence of anaemia was 18.6% (n=448). However, there were only 407 cards found. Most participants were Malays (89.4%), aged 20–40 years (93.6%) and married (96.3%). Almost all anaemia cases (96.5%) were mild (Hb level of 9–10.9 g/dL). Approximately 34.4% of the mothers were already anaemic at booking; 77.6% belonged to the B40 income group; and 31.6% had poor pregnancy spacing of <2 years. Iron deficiency anaemia was the most common type of anaemia (51.0%), followed by dilutional anaemia (34.0%), which did not normalise at 36 weeks of gestation. Anaemia was associated with lower educational (p<0.05) and Hb levels at booking (p<0.001).

**Conclusion:**

Having normal maternal Hb levels in early pregnancy especially at booking is crucial, as it may reduce the possibility of anaemia during pregnancy. Early screening and supplementation of at-risk pregnancies may be applied as a preventive strategy. Suitable methods of iron treatment and investigation need further exploration.

## Introduction

Anaemia in pregnancy remains a public health threat with major health consequences in Malaysia. Earlier research has shown that severe anaemia increases perinatal mortality and morbidity as a result of preterm delivery and intrauterine growth retardation.^1,2^ Approximately 38% (32 million) and 48% of pregnant women are anaemic globally and in South East Asia, respectively.^3^ A local study in Kelantan revealed that 34.6% of pregnant women had anaemia, which is comparable with the global prevalence.^4^

The aetiology of anaemia in pregnancy is multifactorial, and in developing countries such as Malaysia, the most common nutrition-related cause of anaemia is iron deficiency.^5,6^ An earlier review showed that up to 50-80% of women in their reproductive age had anaemia secondary to iron deficiency.^7^ Other causes of maternal anaemia include other micronutrient deficiencies, haemoglobinopathies, parasitic infections and chronic infections.^8,9^

Despite the relatively high documented prevalence of anaemia among antenatal mothers, related data in the local setting remain sparsely available. This study aimed to determine the prevalence of anaemia among antenatal mothers attending health clinics in Alor Gajah, Melaka, and identify the associated risk factors and predictors. The findings are expected to help improve antenatal care in our area.

### Objective

The general objective of the study was to evaluate the prevalence and associated factors of anaemia during pregnancy in Alor Gajah. The specific objectives were to identify the sociodemographic characteristics, possible risk factors of anaemia and possible causes of anaemia among antenatal mothers.

## Methods

### Study setting and design

A retrospective chart review was conducted among all antenatal mothers registered in all 10 health clinics in Alor Gajah in 2018.

### Study population

Antenatal mothers with a haemoglobin (Hb) level of <11.0 g/dL at 36±1 weeks of gestation from 1 January 2018 to 31 December 2018 were included in the study.

### Study criteria

Pregnant mothers who reached 36/52 weeks of gestation (35-38/52 weeks of gestation) at any date in 2018 and were registered in our health clinics were included. Antenatal mothers who attended our clinics for checkup but did not register under our care and antenatal mothers who did not attend our clinics for check-up at 35-38/52 weeks of gestation owing to various reasons were excluded.

### Study instrument

A set of checklist was designed for this study. This checklist consisted of sociodemographic data, pregnancy-related and obstetric risk factors of anaemia, history of chronic diseases and infections that could cause anaemia, history of iron deficiency anaemia (IDA), psychiatric disorders, substance abuse and smoking status.

### Data analysis

Data were analysed using the Statistical Package for the Social Sciences version 23.0.

### Laboratory investigation

The following laboratory parameters were evaluated: Hb level at booking and 36 weeks of gestation; full blood count (FBC) at booking and 36 weeks of gestation; FBP level; BFMP; serum iron level/TIBC; outcomes of ova and cyst in the stool; presence of occult blood in the stool and DNA.

## Results

### Prevalence of anaemia

According to the district annual returns, there were 2,407 antenatal mothers registered in the health clinics in Alor Gajah in 2018. Of them, 448 were identified to have anaemia at 36 weeks gestation, yielding a prevalence of 18.6%. However, only 407 cards were found (90.8%) as in **Table 1**.

### Sociodemographic data

The age of the 407 participants varied from <20 to ≥40 years. Anaemia was more common in the age groups of 20-30 and 30-40 years than in the other age groups. Most women were Malays, were married, completed at least secondary school and were working with a monthly household income ranging from RM 2,113.04 to RM 3,381.35.

There was a significant difference (p<0.05) in the Hb level at 36 weeks of gestation across the different educational level groups. The mean group rank suggested that the participants with a higher educational level had a higher Hb level than those with a lower educational level. Meanwhile, there was no significant difference in the Hb level at 36 weeks of gestation across the different income groups.

**Table 1:** Sociodemographic background of the participants.

Variable	Mean±SD/n (%)
**Household income**	RM 3,381.35±2,113.04
*Age; n=407*	
<20 years	13 (3.2)
20-30 years	187 (45.9)
30-40 years	194 (47.7)
≥40 years	13 (3.2)
*Ethnicity; n=407*	
Malay	364 (89.4)
Chinese	12 (2.9)
Indian	23 (5.7)
Others	8 (2.0)
*Mean±SD/n (%)*	
*Occupation; n=407*	
Employed	219 (53.8)
Unemployed/housewife	188 (46.2)
*Household income; n=370*	
B40	287 (77.6)
M40	82 (22.1)
T20	1 (0.3)
*Educational level; n=392*	
None	1 (0.3)
Primary school	15 (3.8)
Secondary school	220 (56.1)
College/ university	156 (39.8)
*Marital status; n=404*	
Married	389 (96.3)
Unmarried	15 (3.7)

* SD: Standard deviation

### Risk factors

**Table 2** shows various pregnancy-related parameters. Among the known risk factors for anaemia in pregnancy, 31.6% of the pregnant mothers had their last child birth <2 years ago, and only 65.6% showed a normal Hb level at booking. At 36 weeks of gestation, up to 96.5% had mild anaemia at an Hb level between 9 and 11 g/dL.

There was a significant difference (p<0.001) in the Hb level at 36 weeks of gestation across the different Hb level groups at booking. The mean group rank suggested that the participants with a higher Hb level at booking had a higher Hb level at 36 weeks of gestation.

Meanwhile, there was no significant difference (P>0.05) in the Hb level at 36 weeks of gestation across the different categories of age, income and child spacing. The relationship between the Hb levels at 36 weeks of gestation and booking was investigated using Spearman’s rank order correlation (rho). A weak positive correlation was found between the two variables (r=0.26, n=391, p<0.001), with a high Hb level at 36 weeks of gestation associated with a high Hb level at booking. However, the Hb level at 36 weeks of gestation was not associated with age, income and number of gravida.

**Table 2:** Anaemia and pregnancy-related parameters.

Variable	Mean±SD/n (%)
Gravida	2.9±1.7
Hb level at booking	11.4±1.3
WBC at booking	9.1±2.2
RDWSD at booking	11.0±6.8
RDWCV at booking	45.2±5.6
MCH at booking	26.9±4.1
MCV at booking	81.1±12.7
MCHC at booking	32.3±2.2
Hb level at 36±1 weeks of gestation	10.2±0.6
WBC at 36±1 weeks of gestation	9.7±2.2
RDWSD at 36±1 weeks of gestation	12.2±6.4
RDWCV at 36±1 weeks of gestation	47.2±9.8
MCH at 36±1 weeks of gestation	26.3±4.5
MCV at 36±1 weeks of gestation	80.8±12.5
MCHC at 36±1 weeks of gestation	31.1±2.8
Serum iron level	14.2±12.9
Serum TIBC	73.1±14.8
*Tagging colour code; n=407*	
Green	330 (81.1)
Yellow	71 (17.4)
Red	6 (1.5)
*Booking time; n=368*	
≤12 weeks	269 (73.1)
>12 weeks	99 (26.9)
*Last child birth status; n=407*	
<2 years	129 (31.6)
2–5 years	196 (48.1)
>5 years	30 (7.3)
Primid	52 (12.7)
*Thalassemia trait; n=407*	
Yes	39 (9.6)
No	368 (90.4)
*Hb level at booking; n=393*	
<7 g/dL	1 (0.3)
7-9 g/dL	19 (4.8)
9-11 g/dL	115 (29.3)
≥11 g/dL	258 (65.6)
*Hb level at 36±1 weeks of gestation; n=399*	
<7 g/dL	1 (0.3)
7-9 g/dL	13 (3.3)
9-11 g/dL	385 (96.5)
*Stool occult blood; n=7*	
Positive	1 (14.3)
Negative	6 (85.7)
*Stool ova and cyst; n=22*	
Normal	21 (95.5)
Abnormal	1 (4.5)
*BFMP; n=38*	
Positive	0 (0.0)
Negative	38 (100)

### Possible causes

Of the 407 pregnant mothers, only 100 (24.6%) were investigated with peripheral blood films. There were 51% with IDA, 34% with dilutional anaemia due to pregnancy, 6% with thalassemia trait and 3% with South East Asian ovalocytosis. The diagnoses are shown in **Table 3**.

**Table 3:** Diag nosis.

Variable	Mean±SD/n (%)
*Peripheral blood film*	
Performed	100 (24.6)
Not performed	307 (75.4)
*Peripheral blood film diagnosis; n=100*	
Dilutional effect of pregnancy	34 (34.0)
Iron deficiency anaemia	51 (51.0)
South East Asian ovalocytosis	3 (3.0)
Thalassemia trait	6 (6.0)
Others	6 (6.0)

## Discussion

### Sociodemographic data related to anaemia

This study determined the prevalence of anaemia and its association with various sociodemographic and maternal risk factors among the antenatal mothers in Alor Gajah. It also identified the causes of anaemia among the antenatal mothers to implement early and appropriate anaemia intervention.

The prevalence of anaemia was 18.6% (n=448) among the 2,407 antenatal mothers registered in the health clinics in Alor Gajah in 2018. The level projected was substantially low compared with the national prevalence of anaemia of 35%.^10^ In comparison, a cross-sectional study in Singapore showed a prevalence of anaemia of 15.3% at the time of delivery.^11^ Approximately 89.4% (n=364) of the antenatal mothers with anaemia in Alor Gajah were Malays, while 5.7% (n=23) were Indians. This could be attributed to the racial distribution in Alor Gajah, which is dominated by Malays. The finding is supported by the reports of Soh et al. and Hanif et al. in Selangor, demonstrating that the prevalence of anaemia was higher among Malays and Indians.^10,12^

Approximately 93.6% (n=381) of the antenatal mothers who had anaemia were aged between 20 and 40 years, which falls under the reproductive age group. According to a survey conducted by the WHO in 2011, around 30% of women of reproductive age in Malaysia (corresponding to approximately 2 million women) had anaemia.^3^ In contrast, Soh et al. revealed no significant relationship between age and a low Hb level among pregnant mothers in Malaysia.^12^

The majority of our participants had secondary education (56.1%, n=220); only 39.8% (n=156) completed tertiary education. This explicitly shows that antenatal mothers with a higher educational level have higher awareness about the danger of anaemia to their pregnancy than those with a lower educational level; hence, the proportion of affected mothers with a high educational level was small. Soh et al. also reported a similar outcome.^12^

Up to 53.8% (n=219) of our participants were employed compared with 46.2% (n=188) who were housewives. This contradicts an earlier report that working mothers had relatively higher Hb levels, which could be attributed to their higher household income that facilitated better nutrition.^1^ Following this, another study could be conducted to determine the contributing factors of anaemia among working mothers in Alor Gajah.

This study also revealed that the mothers with anaemia had a relatively lower family income. The majority of them had a household income between RM 2,000 and RM 4,000. This is again supported by the report of Soh et al., demonstrating the same association between a low Hb level and low family income.^12^ Mothers from lower-income families are then postulated to have less access to better nutrition and supplementation throughout their pregnancy. Another study further supports the finding that a higher family income was significantly associated with a higher Hb level.^13^

### Pregnancy-related factors associated with anaemia

This study demonstrated that the mean Hb level of the antenatal mothers at booking was 11.4 g/dL. Anaemia was documented towards the late trimester at a mean Hb level of 10.2 g/dL at 36 weeks of gestation. Haniff et al. showed a similar trend in the Hb level as pregnancy progressed.^10^ These findings may well be explained by the physiological expansion of the maternal plasma volume, causing a drop in the Hb level as the gestational age increases.

Most antenatal mothers in this study had their booking at <12 weeks of gestation. Accordingly, the incidence of anaemia remained significant. This could be a result of poor compliance with oral supplementation or inadequate intervention by healthcare providers. An earlier study showed that the rate of compliance with supplementation among antenatal mothers in Malaysia was only 49% as opposed to 80% in a Danish population.^7,14^ The higher rate of compliance among the Danish was attributed to the educational programme of healthcare personnel initiated by their Board of Health.^8^

Other factors contributing to maternal anaemia include parity and spacing between pregnancies. Most patients with anaemia in this study had more than two children and had their last child birth >2 years ago. An African study showed that parous women were more likely to have anaemia than nulliparous women.^15^ The presence of anaemia also increased with gravidity; multigravida women were more likely to be anaemic than primigravida women.

Only a few antenatal mothers were investigated for stool occult blood, stool study for helminths and malaria studies. The numbers of investigated samples were 7, 22 and 38, respectively. Of the samples, one was positive for occult blood and stool study for helminths, while two were positive for malaria infection. Although there were few mothers who had positive results, parasitic infections such as helminths during pregnancy have been reported to be associated with an increased risk of maternal anaemia.^16^

### Diagnosis

Of the 407 antenatal mothers reviewed herein, only 100 had their peripheral blood film sent for investigation. This could be attributed to the lack of awareness among medical officers regarding proper investigation of anaemia in antenatal mothers. Another possible contributing factor could be the perception that iron deficiency is commonly seen as the likely diagnosis, and treatment with iron is often successful without further investigation.

Approximately 51% of our participants were diagnosed with IDA. A study by the University Malaya Medical Centre, Kuala Lumpur, also reported a prevalence of anaemia of 42.5% among their antenatal mothers.^17^ Approximately 65.3% of anaemia cases were classified as IDA with a serum ferritin level of <12 pg/L. In a retrospective study of women from 102 health clinics in Kelantan, the prevalence of anaemia and iron deficiency at booking was as high as 47.5%.^18^ These previous studies showed that both urban and rural areas in Malaysia had a high prevalence of IDA in pregnancy. In addition to the marked increase in the demand of iron, the dilutional effect of pregnancy is also commonly seen in the second trimester when the increment of plasma volume is disproportionate to the production of the red blood corpuscle. This study revealed that up to 34% of antenatal mothers had anaemia due to haemodilution as a result of its physiological adaptation. An earlier study in Nigeria showing that the prevalence of anaemia at booking increases from 26.5% during the first trimester to 56% during the third trimester supports this finding.^19^ Similarly, the study attributed the increased prevalence of mild anaemia to the physiologic haemodilution during pregnancy.

The present study has its limitations, including the incomplete anaemia investigation among the entire studied population. This might have underestimated the possible aetiology of anaemia among the antenatal mothers. In view of the involvement of different clinics, the use of non-standardised machines could also lead to discrepancies in the results. This limitation could be eliminated if the study is performed prospectively in a controlled environment or setting using a standardised machine.

In conclusion, anaemia in pregnancy remains a major concern in Alor Gajah, Melaka. Effort should be geared to early detection of anaemia and prompt treatment prior to delivery to improve the provision of care among antenatal mothers. Medical officers should investigate antenatal mothers timely to identify the aetiology of anaemia apart from initiating routine iron supplementation. Advocacy towards compliance with supplementation and related dietary education should also be included as part of the management plan. The involvement of family members in the routine discussion may offer a better outcome, as mothers would be well supported. These collective efforts could ultimately result in a better management of antenatal mothers and prevent anaemia in future pregnancies.

## Data sharing statement

Data are uploaded in publicly available databases.

**How does this paper make a difference in general practice?**

- This study focused on the associated factors of anaemia among antenatal mothers in Alor Gajah. The findings may help improve the management of antenatal mothers not only in Alor Gajah but also in other places.
